# Lactate administration does not affect denervation‐induced loss of mitochondrial content and muscle mass in mice

**DOI:** 10.1002/2211-5463.13293

**Published:** 2021-09-20

**Authors:** Kenya Takahashi, Yu Kitaoka, Yutaka Matsunaga, Hideo Hatta

**Affiliations:** ^1^ Department of Sports Sciences The University of Tokyo Meguro‐ku Japan; ^2^ Department of Human Sciences Kanagawa University Yokohama Japan

**Keywords:** denervation, lactate, mitochondria, skeletal muscle

## Abstract

Lactate is considered to be a signaling molecule that induces mitochondrial adaptation and muscle hypertrophy. The purpose of this study was to examine whether lactate administration attenuates denervation‐induced loss of mitochondrial content and muscle mass. Eight‐week‐old male Institute of Cancer Research mice underwent unilateral sciatic nerve transection surgery. The contralateral hindlimb served as a sham‐operated control. From the day of surgery, mice were injected intraperitoneally with PBS or sodium lactate (equivalent to 1 g·kg^−1^ body weight) once daily for 9 days. After 10 days of denervation, gastrocnemius muscle weight decreased to a similar extent in both the PBS‐ and lactate‐injected groups. Denervation significantly decreased mitochondrial enzyme activity, protein content, and MCT4 protein content in the gastrocnemius muscle. However, lactate administration did not have any significant effects. The current observations suggest that daily lactate administration for 9 days does not affect denervation‐induced loss of mitochondrial content and muscle mass.

## Graphical abstract

Lactate is considered to be a signaling molecule that induces mitochondrial adaptation and muscle hypertrophy. This study examined effects of lactate administration on denervation‐induced loss of mitochondrial content and skeletal muscle mass. The results showed that neither mitochondrial content nor skeletal muscle mass during 10 days of denervation was changed by lactate administration.

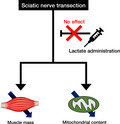

**Abbreviations**
ATP5A: ATP synthase, H^+^ transporting, mitochondrial F1 complex, α‐subunit
BCA: bicinchoninic acid
COX: cytochrome *c* oxidase
CS: citrate synthase
Drp1: dynamin‐related protein 1
ERK: extracellular signal‐regulated kinase
Fis1: mitochondrial fission 1 protein
ICR: Institute of Cancer Research
MCT: monocarboxylate transporter
MEK: mitogen‐activated protein kinase kinase
Mfn2: mitofusin‐2
MTCO1: mitochondrially encoded cytochrome *c* oxidase I
mTOR: mechanistic target of rapamycin
NDUFB8: NADH dehydrogenase (ubiquinone) 1β subcomplex 8
Opa1: optic atrophy 1
p70S6K: ribosomal protein S6 kinase
PGC‐1α: peroxisome proliferator‐activated receptor gamma coactivator 1‐alpha
SDHB: succinate dehydrogenase complex subunit B
UQCRC2: ubiquinol-cytochrome *c* reductase core protein II
β‐HAD: β‐hydroxyacyl‐CoA dehydrogenase

Several situations, such as recovery from injury and illness, necessitate certain periods of muscle disuse, which results in muscle atrophy and negative physical consequences, such as impaired functional muscle strength [1], reduced insulin sensitivity [2], and a decline in basal metabolic rate [3]. Along with muscle wasting, a decline in mitochondrial oxidative capacity and changes in mitochondrial dynamics occur [4, 5]. Previous studies have shown that mitochondria are involved in the regulation of skeletal muscle mass and function [6, 7], suggesting that maintaining mitochondrial content and dynamics during muscle disuse may represent a therapeutic approach for attenuating disuse‐induced muscle wasting.

Lactate is recognized not only as a metabolic intermediate but also as a signaling molecule that induces mitochondrial adaptations [8, 9]. We previously reported that mitochondrial enzyme activity, a biomarker of mitochondrial content, in mouse skeletal muscle increased after lactate administration [10, 11]. Lactate is also assumed to induce muscle hypertrophy. Ohno *et al*. have reported that oral lactate ingestion induced hypertrophy in both the intact and regenerating skeletal muscles of mice [12] and that lactate treatment enhanced the phosphorylation of mTORC1, ERK, and MEK proteins, which are involved in muscle protein synthesis, in C2C12 myotubes [13, 14]. These observations allowed us to hypothesize that daily lactate administration might attenuate muscle disuse‐induced mitochondrial reduction and muscle wasting.

To test this hypothesis, we assessed mitochondrial enzyme activity and proteins following sciatic nerve transection, which is a prevailing animal model of muscle disuse. We also evaluated cellular signaling proteins (mTORC1, p70S6K, ERK, and MEK), which are associated with muscle growth, after a single administration lactate.

## Methods

### Experimental animals

All experiments were approved by the Animal Experimental Committee of The University of Tokyo (no. 27‐14). Male Institute of Cancer Research (ICR) mice (8‐week‐old; CLEA Japan, Tokyo, Japan) were housed individually on a 12:12 h light–dark cycle (dark: 07:00 AM to 07:00 PM) in an air‐conditioned room at 22 °C. Standard chow (MF; Oriental Yeast, Tokyo, Japan) and water were provided *ad libitum* during the experimental period.

### Experiment 1: Effect of lactate administration on denervation‐induced changes in mitochondrial enzyme activity and proteins

Animals were divided into a PBS‐administration group (*n* = 10) and lactate‐administration group (*n* = 10). As described in a section below, all animals underwent sciatic nerve transection surgery on the left hindlimb and sham operation on the right hindlimb, resulting in four subgroups of sham + PBS, denervation + PBS, sham + lactate, and denervation + lactate. From the day of surgery, the animals received PBS or sodium lactate (1 g·kg^−1^ of body weight) via intraperitoneal injection once daily for 9 days. This method of lactate administration was reported in our previous studies to elevate blood lactate concentration to an upper physiological level (˜ 20 mmol·L^−1^) [9]. Twenty‐four hours after the final administration, the gastrocnemius muscle was excised, immediately weighed, rapidly frozen in liquid nitrogen, and stored at −80 °C until further analysis. During the experimental period, food intake was recorded daily. Energy intake was calculated by the addition of calories from food intake (3.59 kcal·g^−1^) and sodium lactate (2.90 kcal·g^−1^).

### Experiment 2: Effect of lactate administration on cellular signaling pathway

Animals without the surgery were used in experiment 2. Animals were allocated to a PBS‐administration control group (CON; *n* = 10) or a lactate‐administration group (LAC; *n* = 11), and received intraperitoneal injections of PBS or sodium lactate (1 g·kg^−1^ of body weight). At 60 min after administration, the gastrocnemius muscle was excised, rapidly frozen in liquid nitrogen, and stored at −80 °C until further analysis.

### Sciatic nerve transection

We used 10 days of unilateral sciatic nerve transection as surgical denervation in the experiment 1, because previous studies showed that this model induces sufficient muscle atrophy and decreases the mitochondrial content in skeletal muscle [15, 16]. Briefly, mice were anesthetized with isoflurane [4% (vol/vol) induction, 3% (vol/vol) maintenance, flow rate 0.5 L·min^−1^]. A small incision was made in the posterior aspect of the left hindlimb to expose the sciatic nerve at the level of the femoral trochanter. The sciatic nerve was excised to a length of at least 5.0 mm using small operating scissors. The skin was closed using a surgical glue. The right hindlimb served as a sham‐operated control.

### Mitochondrial enzyme activity

The maximal activities of CS, β‐HAD, and COX were measured as described elsewhere [10, 17]. Frozen and crushed gastrocnemius muscles were homogenized in 100 times (vol/wt) of 100 mm potassium phosphate buffer using a μT‐01 bead crusher (TAITEC, Saitama, Japan). Maximal enzyme activity was measured spectrophotometrically. The total protein concentration in the homogenates was determined using a BCA protein assay (TaKaRa BIO Inc., Shiga, Japan), with activities normalized to total protein concentration.

### Western blotting

Frozen and crushed gastrocnemius muscles were homogenized in 10 times (vol/wt) of a radio immunoprecipitation assay buffer [50 mm Tris/HCl (pH 7.4), 150 mm NaCl, 0.25% deoxycholic acid, 1% NP‐40, and 1 mm ethylenediaminetetraacetic acid] supplemented with a protease inhibitor cocktail (cOmplete Mini, EDTA‐free; Roche Applied Science, Mannheim, Germany) and a phosphatase inhibitor mixture (PhosSTOP; Roche Applied Science). The homogenates were rotated on ice for 60 min and centrifuged at 1500 ***g*** at 4 °C for 20 min. The total protein content of the samples was determined using a BCA protein assay kit (TaKaRa Bio Inc.). Equal amounts of protein, depending on the protein of interest, were loaded onto 7.5% sodium dodecyl sulfate‐polyacrylamide gels and separated by electrophoresis. Proteins were transferred to polyvinylidene difluoride membranes. Western blotting was performed as described previously [10]. Primary antibodies used in the present study were as follows: PGC‐1α (516557; Millipore, La Jolla, CA, USA), MitoProfile Total OXPHOS Rodent WB Antibody Cocktail (NDUFB8, SDHB, UQCRC2, MTCO1, and ATP5A; ab110413; Abcam, Cambridge, UK), COXIV (ab14744; Abcam), CS (ab129095; Abcam), Fis1 (ab96764, Abcam), Drp1 (ab56788, Abcam), Mfn2 (ab124773, Abcam), Opa1 (#612606; BD Transduction Laboratories, Tokyo, Japan), phosphorylated (p‐) mTORSer2448 [#2481; Cell Signaling Technology (CST) Japan, Tokyo, Japan], mTOR (#2983; CST Japan), p‐p70S6KThr389 (#9205; CST Japan), p70S6K (#9202; CST Japan), p‐ERKThr202/Tyr204 (#4370; CST Japan), ERK (#4695; CST Japan), p‐MEKSer217/Ser221 (#9154; CST Japan), and MEK (#8727; CST Japan). Antibodies (Qiagen, Tokyo, Japan) against MCT1 and MCT4 were raised in rabbits against the C‐terminal region of each MCT and were used in our previous studies [18, 19, 20]. The following secondary antibodies were used in the current study: rabbit anti‐goat (H&L) (A102PT; American Qualex, San Clemente, CA, USA) and mouse anti‐goat (H&L) (A106PU; American Qualex). Blots were scanned and quantified using ChemiDoc XRS (Bio‐Rad Laboratories, Hercules, CA, USA) and quantity one (v.4.5.2; Bio‐Rad). Ponceau staining was used to verify consistency in gel loading.

### Statistical analysis

All data are presented as means ± standard error of the means (SEM). Student's *t*‐test or two‐way ANOVA (denervation × lactate) was performed using graphpad prism (v.9.0, Macintosh, GraphPad Software, La Jolla, CA, USA). Statistical significance was defined as *P* < 0.05.

## Results

### Experiment 1: Effect of lactate administration on denervation‐induced changes in mitochondrial enzyme activity and proteins

**Fig. 1:**
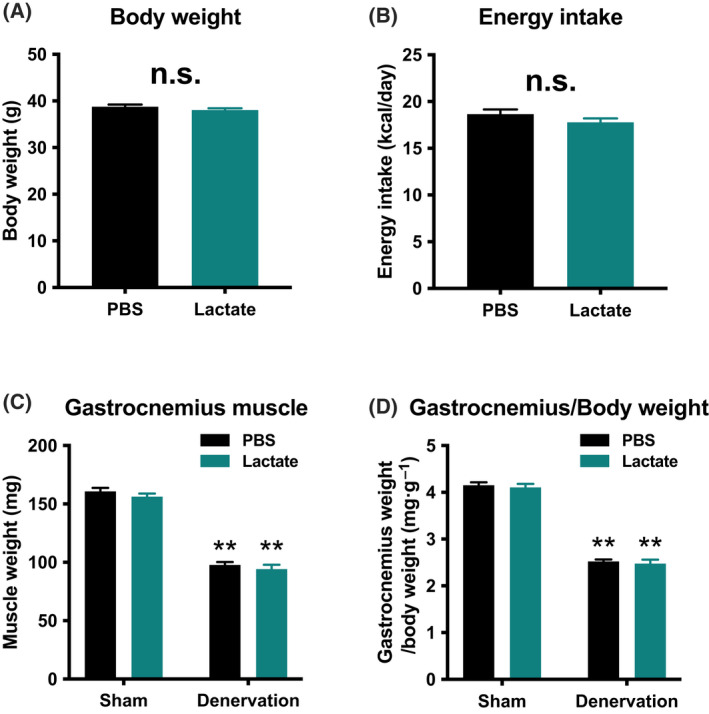
Animal characteristics. Body weight (A), energy intake (B), gastrocnemius muscle weight (C), and relative gastrocnemius weight to body weight (D) in the Experiment 1. Data are presented as mean ± SEM (*n* = 10). Student's *t*‐test or two‐way ANOVA (denervation × lactate administration) was used for statistical evaluation. n.s., not significant. **P < 0.01: main effect of denervation.

**Fig. 2:**
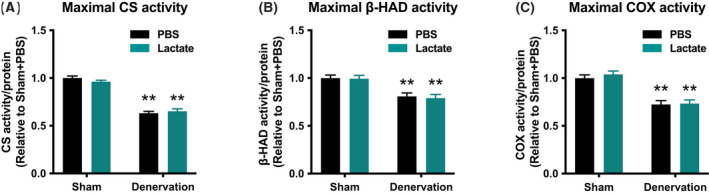
Mitochondrial enzyme activities. Maximal activities of CS (A), β‐HAD (B), and COX (C) in the gastrocnemius muscle (Experiment 1). Data are presented as means ± SEM (*n* = 10). Two‐way ANOVA (denervation × lactate administration) was used for statistical evaluation. **P < 0.01: main effect of denervation.

**Fig. 3:**
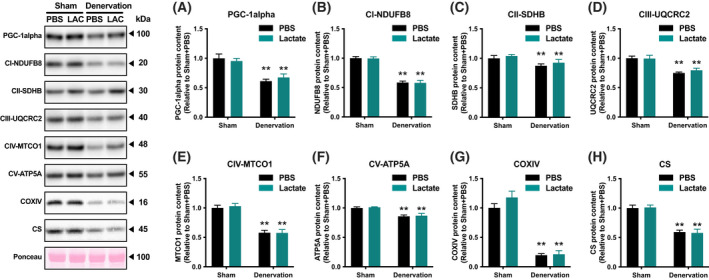
Mitochondrial protein contents. Protein contents of PGC‐1α (A), mitochondrial respiratory chain subunits (B–G), and CS (H) in the gastrocnemius muscle (Experiment 1). Data are presented as means ± SEM (*n* = 10). Two‐way ANOVA (denervation × lactate administration) was used for statistical evaluation. **P < 0.01: main effect of denervation.

**Fig. 4:**
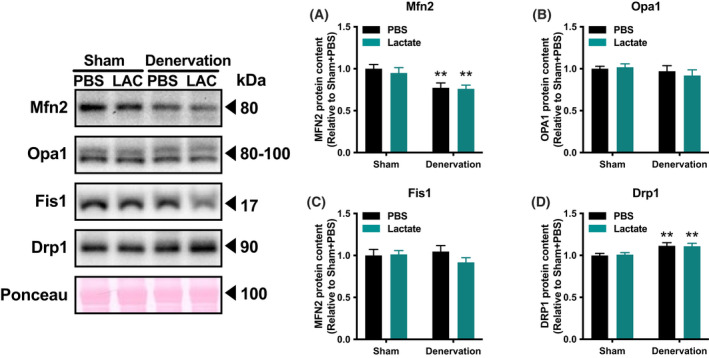
Protein content of mitochondrial dynamics. Protein contents of Mfn2 (A), Opa1 (B), Fis1 (C), and Drp1 (D) in the gastrocnemius muscle (Experiment 1). Two‐way ANOVA (denervation × lactate administration) was used for statistical evaluation. Data are presented as means ± SEM (*n* = 10). **P < 0.01: main effect of denervation.

**Fig. 5:**
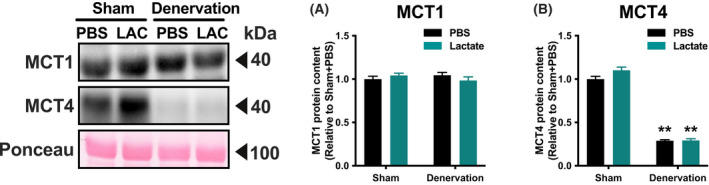
MCT protein contents. Protein contents of MCT1 (A) and MCT4 (B) in the gastrocnemius muscle (Experiment 1). Two‐way ANOVA (denervation × lactate administration) was used for statistical evaluation. Data are presented as means ± SEM (*n* = 10). **P < 0.01: main effect of denervation.

To evaluate the effects of lactate administration in atrophy‐related denervation, we administrated lactate or PBS intraperitoneally for 9 days. There were no differences in mouse final body weight (Fig. 1A) or energy intake (Fig. 1B) between the PBS‐administration group and lactate‐administration group. Although denervation significantly decreased gastrocnemius muscle weight (*P* < 0.01), lactate administration had no such effects (Fig. 1C,D). These observations suggest that no effects of lactate administration in atrophy prevention. To clarify whether lactate administration attenuates denervation‐induced decline in mitochondrial content, we examined activities of key enzymes located within mitochondria. Maximal activity of CS, a biomarker of mitochondrial content, significantly decreased by denervation (*P* < 0.01; Fig. 2A). Additionally, maximal activities of β‐HAD, a rate‐limiting enzyme for fatty acid oxidation, and COX, a rate‐limiting factor for electron transport, also decreased by denervation (*P* < 0.01; Fig. 2B,C). However, no effects of lactate administration were observed in these enzyme activities. To further explore the effect of lactate administration, we evaluated protein content related to mitochondria. Denervation significantly decreased protein content of PGC‐1α, which is known as a master regulator of mitochondrial adaptations (*P* < 0.01; Fig. 3A). Similarly, mitochondrial protein content of NDUFB8, SDHB, UQCRC2, MTCO1, ATP5A, COXIV, and CS was significantly decreased by denervation (*P* < 0.01; Fig. 3B–H). However, lactate administration had no effects on these protein contents, suggesting that lactate administration does not reduce denervation‐induced decrease in mitochondrial content. Next, to investigate the possible effects of intraperitoneal administration of lactate on mitochondria dynamics, we also assessed protein contents that regulate mitochondrial morphology. Denervation decreased levels of the mitochondrial fusion protein Mfn2 (*P* < 0.01; Fig. 4A) and increased fission protein Drp1 (*P* < 0.01; Fig. 4D). No difference was found in Fis1 or Opa1 protein levels (Fig. 4B,C). These results implicit that lactate administration does not alter mitochondrial morphology. Because increases in lactate transport protein content are reported to be associated with an elevated blood lactate concentration, we measured MCT1 and MCT4 protein content. Denervation or lactate administration had no effect on MCT1 protein levels (Fig. 5A). While denervation decreased MCT4 protein content (*P* < 0.01; Fig. 5B), lactate administration had no effects. These suggest that changes in MCT protein contents are not associated with an increase in blood lactate concentration.

### Experiment 2: Effect of lactate administration on cellular signaling pathways

**Fig. 6:**
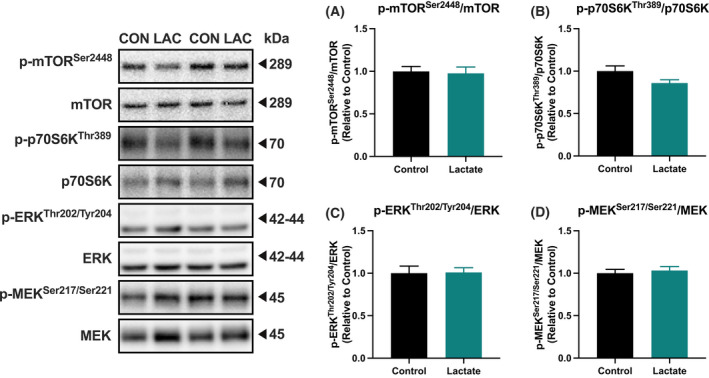
Protein phosphorylation. Phosphorylation status of mTOR (A), p70S6K (B), ERK (C), and MEK (D) in the gastrocnemius muscle at 60 min after lactate administration (Experiment 2). Data are presented as means ± SEM (*n* = 10 or 11). Student's *t*‐test was used for statistical evaluation.

Although previous studies reported that lactate enhanced mTOR/p70S6K and ERK/MEK pathways in C2C12 cells, whether lactate administration activates these signaling pathways in mouse skeletal muscle remains to be examined. For this reason, we evaluated protein phosphorylation of mTOR, p70S6K, ERK, and MEK proteins 60 min after a single administration of lactate in gastrocnemius muscle without denervation. The phosphorylation status of mTOR, p70S6K, ERK, and MEK did not differ significantly (Fig. 6A–D), suggesting that lactate administration does not activate anabolic signaling in skeletal muscle.

## Discussion

Although lactate is considered to be a signaling molecule that induces mitochondrial adaptations [8, 9], the effects of lactate administration during muscle disuse remain to be elucidated. In the current study, we examined whether daily lactate administration reduced denervation‐induced loss of mitochondrial content. Our results showed that lactate administration for 9 days did not affect mitochondrial enzyme activity or protein content in either innervated or denervated gastrocnemius muscle. This is contradictory to our previous studies reporting that mitochondrial enzyme activity in mouse skeletal muscle increased after lactate administration for 3 [10] and 4 weeks [11]. We previously reported that a single lactate administration enhanced PGC‐1α mRNA level in mouse skeletal muscle [9]. Another study reported that mitochondrial adaptation appears to result from the cumulative effects of repetitive increases in mRNAs, including PGC‐1α [21]. The experimental period used in the present study was shorter than that used in our previous studies [10, 11]. Therefore, we assume that lactate administration for 9 days is not sufficient to increase or preserve mitochondrial enzyme activity and protein content in the gastrocnemius muscle.

Mitochondria exist as dynamic networks that are continuously remodeled through fusion and fission. In general, muscle disuse shifts mitochondrial dynamics from fusion to fission to remove dysfunctional mitochondria [7]. In agreement with this, we observed that denervation decreased levels of Mfn2 (a fusion‐associated protein) and increased Drp1 (a fission‐associated protein) abundance. However, lactate administration did not alter the content of proteins involved in mitochondrial dynamics, suggesting that lactate administration does not affect mitochondrial dynamics, at least after 9 days of lactate administration.

It is worth noting that previous studies have reported that mitochondrial oxygen consumption rates are altered without differences or changes in mitochondrial content and dynamics [22, 23, 24]. Thus, we cannot rule out the possibility that lactate administration altered mitochondrial respiratory function without observable effects on enzyme activity and protein content in the present study.

In addition to mitochondrial adaptation, lactate has been assumed to exert an anabolic effect on skeletal muscle [25, 26, 27]. This is based on the observation that low‐load resistance exercise with blood flow restriction, which increases blood lactate levels, has been shown to increase muscle mass to a greater extent compared with repetition‐matched low‐load resistance exercise without blood flow restriction [28]. In the present study, neither innervated nor denervated muscle weights changed after lactate administration. In accord with this, we previously reported no difference in muscle mass after lactate administration [10, 11]. Although a previous study showed that co‐ingestion of lactate and caffeine prior to endurance exercise training increased skeletal muscle mass of rats to a greater extent compared with endurance exercise training alone [14], the authors did not examine lactate and caffeine separately, raising the possibility that caffeine, rather than lactate, contributed to muscle hypertrophy. Moreover, another study reported no differences in muscle mass of mice after co‐ingestion of lactate and caffeine with voluntary running compared with voluntary running alone [29]. Collectively, it is likely that lactate does not induce skeletal muscle hypertrophy and that vascular occlusion training‐induced muscle growth does not result from elevated blood lactate concentration.

Of note, however, Ohno *et al*. [12] reported that oral lactate administration alone induced hypertrophy both in the intact and in the regenerating muscles. While they used C57BL/6J mice, the present study used ICR mice, as experimental animals. A previous study reported that electrical stimulation‐induced resistance exercise induced skeletal muscle hypertrophy in Sprague Dawley rats, but not in Wistar rats [30], suggesting that responsiveness to stimuli can differ depending on animal strains. Thus, there could be interspecies differences in response to lactate administration.

Based on the observation that lactate enhanced ERK and MEK phosphorylation via GPR81 activation in C2C12 cells [13], Ohno *et al*. [12] posited that lactate‐induced skeletal muscle hypertrophy is dependent on GPR81 activation. Moreover, others reported that lactate treatment enhanced p70S6K phosphorylation in C2C12 cells [14]. However, they did not examine effect of lactate administration on the signaling pathways in skeletal muscle. In the present study, neither skeletal muscle weight nor phosphorylation status of ERK, MEK, p70S6K, and mTOR was not altered by lactate administration in mouse skeletal muscle. Supporting the current observations, a previous study has demonstrated that lactate infusion does not augment resistance exercise‐induced p70S6K, ERK, and MEK phosphorylation or protein synthesis in human skeletal muscle [31]. Although whether there are interspecies differences in GPR81 expression and responses to lactate administration is yet to be investigated, elevated circulating lactate concentrations do not appear to activate anabolic signaling in skeletal muscle.

The transport of lactate across the sarcolemmal membrane is primarily catalyzed by MCT1 and MCT4 [32]. Given that an elevated lactate concentration is considered to increase the MCT protein content [8, 33, 34], we examined whether lactate administration during denervation changed MCT protein content. In the current study, lactate administration did not change MCT protein content in innervated or denervated muscles. We previously reported that MCT protein content did not alter after either 3 weeks of lactate injection [10] or after 4 weeks of lactate ingestion [11]. These findings suggest that lactate does not affect MCT protein content in skeletal muscle, not only in innervated muscles, but also in muscles without contractile activity.

The present observation that MCT4, but not MCT1 protein content, decreased after 10 days of denervation, is in accordance with our previous study [15]. Other studies showed that both MCT1 and MCT4 protein levels decrease together with lactate transport capacity in rat skeletal muscle after 3 weeks of denervation, but not after 3 days of denervation [35, 36]. We assume that 10 days of denervation were insufficient to decrease MCT1 protein content in mouse skeletal muscle. Moreover, we previously reported that moderate‐intensity exercise training maintained MCT1 protein content, but not MCT4 protein levels, in high‐intensity trained equine muscle [34]. Another study reported that MCT4 abundance, but not MCT1, significantly decreased 6 weeks after cessation of sprint interval training [37]. These observations suggest that MCT4 is more sensitive to reductions in muscle contractile activity than MCT1.

Finally, it should be noted that sciatic denervation is a drastic model that induces loss of mitochondrial content and skeletal muscle mass compared with other muscle disuse models, such as hindlimb suspension and cast immobilization [38, 39, 40]. Additionally, we did not evaluate time‐course changes involving mitochondrial content and skeletal muscle weight. Moreover, our data regarding muscle size are limited to absolute and relative weights. Evaluating other variables, including maximum contraction force, fiber cross‐sectional area, and fibrosis area would provide good indices for muscle function and quality. Further studies are clearly needed to clarify the effects of lactate administration on different inactivity models and durations, as well as other skeletal muscle properties.

In summary, lactate administration for 9 days did not attenuate denervation‐induced loss of mitochondrial content or muscle mass. Additionally, a single administration of lactate did not activate the cellular signaling proteins mTORC1, p70S6K, ERK, and MEK, which are involved in muscle protein synthesis. The current observations suggest that 9 days of lactate administration with and without denervation do not affect mitochondrial content and skeletal muscle mass, and do not support the popular belief that lactate activates anabolic signaling and induces skeletal muscle hypertrophy.

## Conflict of interest

The authors declare no conflict of interest.

## Author contributions

KT, YK, and HH conceived and designed the project; KT and YM performed the experiments; KT analyzed data; KT, YK, YM, and HH interpreted the data; KT wrote the manuscript; YK, YM, and HH revised the manuscript; all authors have read and approved the final version of the manuscript.

## Data Availability

The authors state that all data will be available from the corresponding author upon reasonable request.
